# Adipose Stromal Cell-Secretome Counteracts Profibrotic Signals From IPF Lung Matrices

**DOI:** 10.3389/fphar.2021.669037

**Published:** 2021-07-28

**Authors:** Gwenda F. Vasse, Lisette Van Os, Marina De Jager, Marnix R. Jonker, Theo Borghuis, L. Tim Van Den Toorn, Pytrick Jellema, Eric S. White, Patrick Van Rijn, Martin C. Harmsen, Irene H. Heijink, Barbro N. Melgert, Janette K. Burgess

**Affiliations:** ^1^ University of Groningen, University Medical Center Groningen, Department of Biomedical Engineering, Groningen, Netherlands; ^2^ University of Groningen, University Medical Center Groningen, W.J. Kolff Institute for Biomedical Engineering and Materials Science, Groningen, Netherlands; ^3^ University of Groningen, Department of Molecular Pharmacology, Groningen Research Institute for Pharmacy, Groningen, Netherlands; ^4^ University of Groningen, University Medical Center Groningen, Groningen Research Institute for Asthma and COPD (GRIAC), Groningen, Netherlands; ^5^ University of Groningen, University Medical Center Groningen, Department of Pathology and Medical Biology, Groningen, Netherlands; ^6^ Division of Pulmonary and Critical Care Medicine, University of Michigan Medical School, Ann Arbor, MI, United States; ^7^ University of Groningen, University Medical Center Groningen, Department of Pulmonology, Groningen, Netherlands

**Keywords:** idiopathic pulmonary fibrosis (IPF), adipose tissue-derived stromal/stem cells (ASCs), decellularized lung matrices, denatured collagen, collagen hybridizing peptide, extracellular matrix (ECM), primary lung fibroblasts, conditioned medium

## Abstract

**Introduction:** Idiopathic pulmonary fibrosis (IPF) is a fibrotic lung disease characterized by excess deposition and altered structure of extracellular matrix (ECM) in the lungs. The fibrotic ECM is paramount in directing resident cells toward a profibrotic phenotype. Collagens, an important part of the fibrotic ECM, have been shown to be structurally different in IPF. To further understand the disease to develop better treatments, the signals from the ECM that drive fibrosis need to be identified. Adipose tissue-derived stromal cell conditioned medium (ASC-CM) has demonstrated antifibrotic effects in animal studies but has not been tested in human samples yet. In this study, the collagen structural integrity in (fibrotic) lung tissue, its interactions with fibroblasts and effects of ASC-CM treatment hereon were studied.

**Methods:** Native and decellularized lung tissue from patients with IPF and controls were stained for denatured collagen using a collagen hybridizing peptide. Primary lung fibroblasts were seeded into decellularized matrices from IPF and control subjects and cultured for 7 days in the presence or absence of ASC-CM. Reseeded matrices were fixed, stained and analyzed for total tissue deposition and specific protein expression.

**Results:** In both native and decellularized lung tissue, more denatured collagen was observed in IPF tissue compared to control tissue. Upon recellularization with fibroblasts, the presence of denatured collagen was equalized in IPF and control matrices, whereas total ECM was higher in IPF matrices than in the control. Treatment with ASC-CM resulted in less ECM deposition, but did not alter the levels of denatured collagen.

**Discussion:** Our data showed that ASC-CM can inhibit fibrotic ECM-induced profibrotic behavior of fibroblasts. This process was independent of collagen structural integrity. Our findings open up new avenues for ASC-CM to be explored as treatment for IPF.

## Introduction

Idiopathic pulmonary fibrosis (IPF) is a progressive and devastating lung disease with an estimated survival of less than 5 years after diagnosis. It is mainly characterized by excessive and imbalanced accumulation of extracellular matrix (ECM) proteins in the interstitium of lung tissue (Lederer and Martinez, 2018). ECM is a highly dynamic structure that is deposited and constantly remodeled by surrounding cells with fibroblasts and myofibroblasts being the most important producers of ECM proteins in the lung (Burgess et al., 2016; Burgstaller et al., 2017). In IPF, mainly myofibroblasts contribute to the dense ECM in characteristic fibroblastic foci (Raghu et al., 2011). Formation of these fibroblastic foci disrupts blood flow to alveolar septa, which leads to alveolar septa damage and thus a decreased gas exchange capacity (Yamaguchi et al., 2017).

Multiple ECM proteins such as fibronectin and periostin are known to be more abundant in lung tissue of patients with IPF (Naik et al., 2012; Lehtonen et al., 2016; Tomos et al., 2017; Kudo and Kii, 2018), but collagens are the most abundant. It is becoming increasingly evident that not only the amount, but also the folding and structural integrity of collagen, is different in fibrosis (Kristensen et al., 2014; Ricard-Blum et al., 2018). Collagen molecules are triple helical protein structures that can, depending on the fibrillar or non-fibrillar type of collagen, assemble into collagen fibrils and larger collagen fibers (Ricard-Blum, 2011; Chang and Buehler, 2014). In lung tissue from patients with IPF, collagen fibers are altered structurally: the fibers are thicker and more mature/organized as compared to collagen fibers in control lung tissue (Tjin et al., 2017). The recent introduction of a collagen hybridizing peptide has made advanced imaging of collagen remodeling on a molecular level possible (Li and Yu, 2013). This peptide specifically binds to a repeating triple-helical motif that is shared by all 28 collagen types. Upon collagen denaturation and/or partial degradation, improper folding of the triple helix exposes the triple-helical motif and allows the labeled peptide to hybridize with the denatured or structurally disrupted collagen (Li and Yu, 2013; Hwang et al., 2017a). Using this collagen hybridizing peptide, augmented levels of structurally disrupted collagen have been observed in lung tissue of mice with bleomycin-induced pulmonary fibrosis (Hwang et al., 2017a), but it is still unclear whether these molecular changes are also present in lung tissue from patients with IPF and whether these changes play a notable role in fibrotic responses.

Currently, only two drugs have been approved to treat IPF: pirfenidone and nintedanib. Although these two drugs can slow down disease progression in some patients, they do not improve long-term mortality and patients can suffer from severe side-effects (Spagnolo et al., 2015). As an alternative or addition to pharmacological approaches, stem cell-based therapies are being explored for the treatment of IPF. Adipose tissue-derived stromal cells (ASCs) have been tested experimentally for the treatment of various conditions, including IPF (Barczyk et al., 2015). A variety of studies showed attenuated, but not resolved, fibrosis after ASC treatment in bleomycin-induced lung fibrosis in mice and rats (Lee et al., 2014; Jiang et al., 2015; Tashiro et al., 2015; Rathinasabapathy et al., 2016; Reddy et al., 2016; Llontop et al., 2018; Felix et al., 2020). Similar results were achieved in other rat models of pulmonary fibrosis (Fikry et al., 2015; Chen et al., 2018; Zhang et al., 2019; Radwan et al., 2020). A phase Ib, non-randomized clinical trial in patients with IPF has shown that even though administration of ASCs was safe and hinted toward advantageous short-term effects, the long-term follow-up showed unaltered disease progression and survival rates (Tzouvelekis et al., 2013; Ntolios et al., 2018). Interestingly, the regenerative effect of ASCs lies not only with their differentiation capacity, but mostly in the soluble factors they produce (Salgado et al., 2010). The secretome of ASCs consists of various molecules involved in tissue regeneration, apoptosis, host cell proliferation and angiogenesis (Salgado et al., 2010; Blaber et al., 2012; Sabin and Kikyo, 2015). ASC-derived culture medium, also called ASC-conditioned medium (ASC-CM), can be used to administer only this secretome. The use of ASC-CM as opposed to ASCs themselves prevents any potential harmful effects of the ASCs, as ASCs respond to their environment and could react detrimentally in a profibrotic environment (Yan et al., 2007; Liu et al., 2018). ASC-CM has been shown to reduce lung injury in a bleomycin-induced pulmonary fibrosis rat model (Rathinasabapathy et al., 2016; Felix et al., 2020) and could therefore be an alternative for ASC treatment in IPF. Yet, to date no research has been reported on the possible modulating effect of ASC-CM in human (fibrotic) lungs.

Decellularized matrices can be employed to study the influence of human (fibrotic) lung ECM on cellular behavior, as well as the impact of soluble factors on these cell-matrix interactions. The decellularized matrices can be obtained by treating lungs with various chemicals in a multistep process, removing all cells but retaining the majority of the ECM composition and structure (Booth et al., 2012). Subsequent culturing of freshly isolated primary human lung cells in these scaffolds can provide important insights in the response and matrix remodeling behavior of these cells. Previously, it has been shown that IPF-derived decellularized matrices direct healthy lung fibroblasts toward a profibrotic phenotype (Booth et al., 2012; Parker et al., 2014), emphasizing the ability of ECM to modulate cell behavior and the importance of this constant two-way interaction in the fibrotic response.

In this study, we characterized collagen structural integrity in native and decellularized human lung tissue from control patients and patients with IPF, hypothesizing that collagen structural organization is more disrupted in IPF lungs compared to control lungs. Furthermore, primary human lung fibroblasts were seeded into control and IPF-derived decellularized lung matrices to study cell-ECM crosstalk in the presence or absence of ASC-derived conditioned medium. We hypothesized that upon recellularization, fibroblasts would be driven toward profibrotic behavior in the IPF lung ECM as opposed to the control lung ECM and that administration of ASC-CM would impair the fibrotic process.

## Materials and Methods

### Human Lung Tissue

Human fibrotic lung tissue was collected with informed consent from patients with interstitial lung disease (ILD) undergoing lung transplantation at either the University Medical Center Groningen (UMCG) or at the Erasmus Medical Center Rotterdam. Human non-fibrotic control lung tissue was obtained at the UMCG from patients undergoing surgical resection for carcinoma. In tumor resections, histologically normal lung tissue was taken as far distally as possible from the tumor and assessed visually by a pathologist for the absence of abnormalities with a standard haematoxylin and eosin staining. All available donor characteristics are listed in Table 1. In Groningen, the study protocol was consistent with the Research Code of the University Medical Center Groningen (www.umcg.nl/EN/Research/Researchers/General/ResearchCode/Paginas/default.aspx) and the Dutch national ethical and professional guidelines (“Code of conduct; Dutch federation of biomedical scientific societies”; http://www.federa.org). The Medical Ethical Committee of the Erasmus Medical Center Rotterdam approved all protocols followed in that center.

**TABLE 1:** Overview of lung tissue and lung fibroblast donor characteristics.

Donor	Diagnosis	Material	Gender	Age	Smoking status
1	x	Control fibroblasts	Male	65	Ex-smoker
2	x	Control fibroblasts	Female	65	Non-smoker
3	x	Control fibroblasts	Male	50	Non-smoker
4–7	N.A.	Control decellularized lung matrix	N.A.	N.A.	N.A.
8–11	IPF	IPF decellularized lung matrix	N.A.	N.A.	N.A.
12	NSIP	Fibrotic lung tissue	Male	61	Non-smoker
13	End-stage fibrosis NOS/NSIP	Fibrotic lung tissue	Male	53	Non-smoker
14	IPF	Fibrotic lung tissue	Male	61	Ex-smoker
15	IPF	Fibrotic lung tissue	Male	64	Non-smoker
16	IPF	Fibrotic lung tissue	Male	61	Ex-smoker
17	N.A.	Fibrotic lung tissue	N.A.	48	N.A.
18	Scleroderma-associated pulmonary fibrosis	Fibrotic lung tissue	N.A.	45	N.A.
19	Non-IPF	Fibrotic lung tissue	Male	N.A.	N.A.
20	Methotrexate induced pulmonary fibrosis	Fibrotic lung tissue	Male	61	N.A.
21	Scleroderma-associated pulmonary fibrosis	Fibrotic lung tissue	N.A.	N.A.	N.A.
22	N.A.	Fibrotic lung tissue	N.A.	N.A.	N.A.
23	Polymyositis-associated fibrosis	Fibrotic lung tissue	N.A.	N.A.	N.A.
24	IPF	Fibrotic lung tissue	Male	42	N.A.
25	SFTPA2 mutation-associated pulmonary fibrosis	Fibrotic lung tissue	Female	27	N.A.
26	x	Control lung tissue	N.A.	N.A.	N.A.
27	x	Control lung tissue	N.A.	N.A.	N.A.
28	x	Control lung tissue	N.A.	N.A.	N.A.
29	x	Control lung tissue	N.A.	N.A.	N.A.

Human lung tissue for decellularization was obtained from non-transplantable donors (control, non-fibrotic) and from explants of patients with IPF receiving a lung transplant at the University of Michigan Medical Center. These approaches were deemed exempt from oversight by The University of Michigan Institutional Review Board, as all tissues were de-identified and coming from deceased donors.

### Sample Preparation

Pieces of native lung tissue were fixed in formalin, embedded in paraffin and cut into 5 µm sections using standard procedures. Prior to staining, sections were deparaffinized also using standard procedures. Decellularized lung tissue was prepared as described previously (Booth et al., 2012) and stored in PBS containing 1% penicillin-streptomycin (Gibco Laboratories, Grand Island, NE, United States) at 4°C. Decellularized and recellularized matrices (see below for details) were fixed, embedded in paraffin and sectioned at 4 µm for staining.

### Cell Culture

Primary human lung fibroblasts were isolated from lung tissue of control individuals at the UMCG as described earlier (Krimmer et al., 2012). Cells were derived from three donor tissues. An overview of the donor characteristics is provided in Table 1. The fibroblasts were cultured in low glucose Dulbecco’s Modified Eagle Medium (DMEM, Lonza, Basel, Switzerland) supplemented with 10% fetal bovine serum (FBS, Sigma, St. Louis, MI, United States), 100 U/ml penicillin, 100 µg/ml streptomycin (Pen/Strep, Gibco, Waltham, MA, United States) and 4 mM L-glutamine (Lonza, Basel, Switzerland). Cells were maintained in an incubator at 37°C and 5% CO_2_.

Adipose tissue-derived stromal cells (ASCs) were isolated from liposuction-derived fat tissue as described before (Przybyt et al., 2013) and grown to confluency in the above-described supplemented DMEM in an incubator at 37°C and 5% CO_2_. At confluency, ASC culture medium was changed to low glucose DMEM supplemented with 0.1% bovine serum albumin (BSA, A8806-5g, Sigma, St Louis, United States), 100 U/mL penicillin, 100 µg/ml streptomycin and 4 mM L-glutamine. Then, ASC conditioned media (ASC-CM) was prepared as reported previously (Spiekman et al., 2014). In short, media was aspirated from a confluent ASC layer after 24 h incubation, centrifuged and filtered using a 0.22 µm filter. ASC-CM was derived from cells below passage 8. ASC-CM was stored at −20°C until further use.

### Matrix Recellularization

Primary human lung fibroblasts (Table 1) were seeded in IPF and control decellularized lung matrices by incubating all matrices together with the fibroblasts on a rotary mixer at 37°C for 24 h, at a concentration of 250.000 cells/matrix in low glucose DMEM supplemented with 0.1% BSA, 100 U/ml penicillin, 100 µg/ml streptomycin and 4 mM L-glutamine (further referred to as basal medium). Recellularized matrices were then separated for further experiments. The recellularized matrices were subsequently incubated in basal medium or in a 1:1 mix of basal medium and ASC-derived conditioned medium (ASC-CM) at 37°C and 5% CO_2_. As we sought to investigate the effects of the fibrotic ECM and ASC-CM on fibroblast behavior in this study, FBS was omitted to prevent it from activating the fibroblasts and possibly masking any effects from the ECM or the ASC-CM on the cells. After 7 and 14 days, the recellularized matrices were formalin fixed and paraffin embedded for further analysis.

### CHP Staining

Sections of native lung tissue and pieces of decellularized lung tissue were stained using collagen hybridizing peptide-Cy3 conjugate (R-CHP), according to the manufacturer’s protocol (3Helix Inc., Salt Lake City, UT, United States). A working solution of 5 μM R-CHP in PBS was heated at 80°C for 5 min and subsequently cooled down in an ice bath for 15–90 s to prevent heat shock to the lung tissue upon addition. After adding the R-CHP working solution, the lung tissues were incubated overnight at 4°C. Decellularized lung tissues were incubated while gently shaking. After incubation, the samples were washed three times in PBS for 5 min (lung tissue sections) or 30 min (decellularized lung tissue). Decellularized lung tissue was subsequently kept in PBS and lung tissue sections were mounted using DePeX mounting medium (Serva, Heidelberg, Germany). Both sample types were stored at 4°C until further analysis.

Sections of recellularized matrices were stained using collagen hybridizing peptide-biotin conjugate (B-CHP), according to the manufacturer’s instructions (3Helix Inc.). Endogenous peroxidases were blocked by incubating the slides for 30 min in PBS with 0.3% H_2_O_2_. The slides were then washed three times in PBS, followed by removal of endogenous biotin and avidin binding site using an Avidin/Biotin blocking kit (Agilent, Santa Clara, CA, United States). Subsequently, a working solution of 0.02 μM B-CHP was prepared and added to the slides as described above for R-CHP. After overnight incubation at 4°C and three PBS washes, the slides were incubated with streptavidin peroxidase (Agilent) (1:300) in PBS with 1% BSA and 2% human AB serum (Sigma-Aldrich, Zwijndrecht, Netherlands) for 1 h. After a PBS wash, color was developed by incubating 10 min with NovaRED (Vector Labs, Burlingame, CA, United States) followed by three washes in demi water and an eosin counterstain for 2 min. After dehydration, the slides were mounted in Tissue-Tek Coverslipping Film with the Sakura Tissue-Tek Film Coverslipper (Sakura, Japan).

### Histology

Sections of native lung tissue and decellularized matrices were stained to visualize total collagen using a Masson’s trichrome stain kit (Sigma-Aldrich) according to the instructions of the manufacturer. Sections of recellularized matrices were stained with haematoxylin and eosin staining through consecutive incubation in the following solutions at room temperature: haematoxylin (Merck, Kenilworth, United States) for 5 min, regular tap water for 5 min, eosin (Chroma, Bellows Falls, United States) for 2 min, washed twice in 96% ethanol and twice in 100% ethanol. After staining, the dehydrated slides were mounted in Tissue-Tek Coverslipping Film with the Sakura Tissue-Tek Film Coverslipper.

### Microscopy and Image Analysis

R-CHP staining in decellularized lung tissue and native tissue sections was imaged and captured using a Leica SP2 confocal microscope. Per subject, at least three fields of view were acquired from one experimental replicate. Lung tissue had to cover more than 50% of the image, with focus on alveolar regions while avoiding large airways and blood vessels. Decellularized lung tissue had to cover 30% of the image. The mean grey value of each image was analyzed using Fiji (Schindelin et al., 2012). Masson’s trichrome, H&E and B-CHP staining were imaged and captured using a NanoZoomer XR digital slide scanner (Hamamatsu, Japan). Whole sections were imaged and data were generated from all pixels in these images. Representative images in the figures show a magnified part of the whole section. The density and distribution of the stainings were quantified automated using Fiji. Color deconvolution vectors were optimized to separate double stainings accurately (Ruifrok et al., 2003). Total tissue surface area was measured in grayscale images. The number of pixels above the threshold was used to calculate the positive staining area, while the average staining intensity was based on the intensity of these pixels above the threshold. Thresholds were set to an intensity value where background is excluded. The total positive staining above the threshold was divided in three equal categories (weak, moderate and strong) according to the pixel intensities. Weak, moderate and strong intensity was calculated as a percentage of the total positive area. For the Masson Trichrome staining, additional quantification of the relative amount of collagen in the total image area was carried out by dividing the number of collagen-positive pixels by the total number of pixels within the image area. Images were processed in batches using macros. An overview of the image analysis process can be found in Supplementary Figure 1.

### Statistical Analyses

Normality of the datasets was tested with a QQ plot as reported by Morgan (2017) and a Shapiro-Wilk test. QQ plots can be found in Supplementary Figure 2. When normality was confirmed, the data were tested with an unpaired *t*-test or a two-way ANOVA with a post hoc test controlling for the false discovery rate (original FDR method of Benjamini and Hochberg). *p* < 0.05 was considered significant. Statistical analyses were performed in GraphPad Prism 8.0 (GraphPad Software, La Jolla, CA, United States).

## Results

### Higher Levels of Denatured Collagen in Lung Tissue From Patients With Idiopathic Pulmonary Fibrosis

**FIGURE 1:**
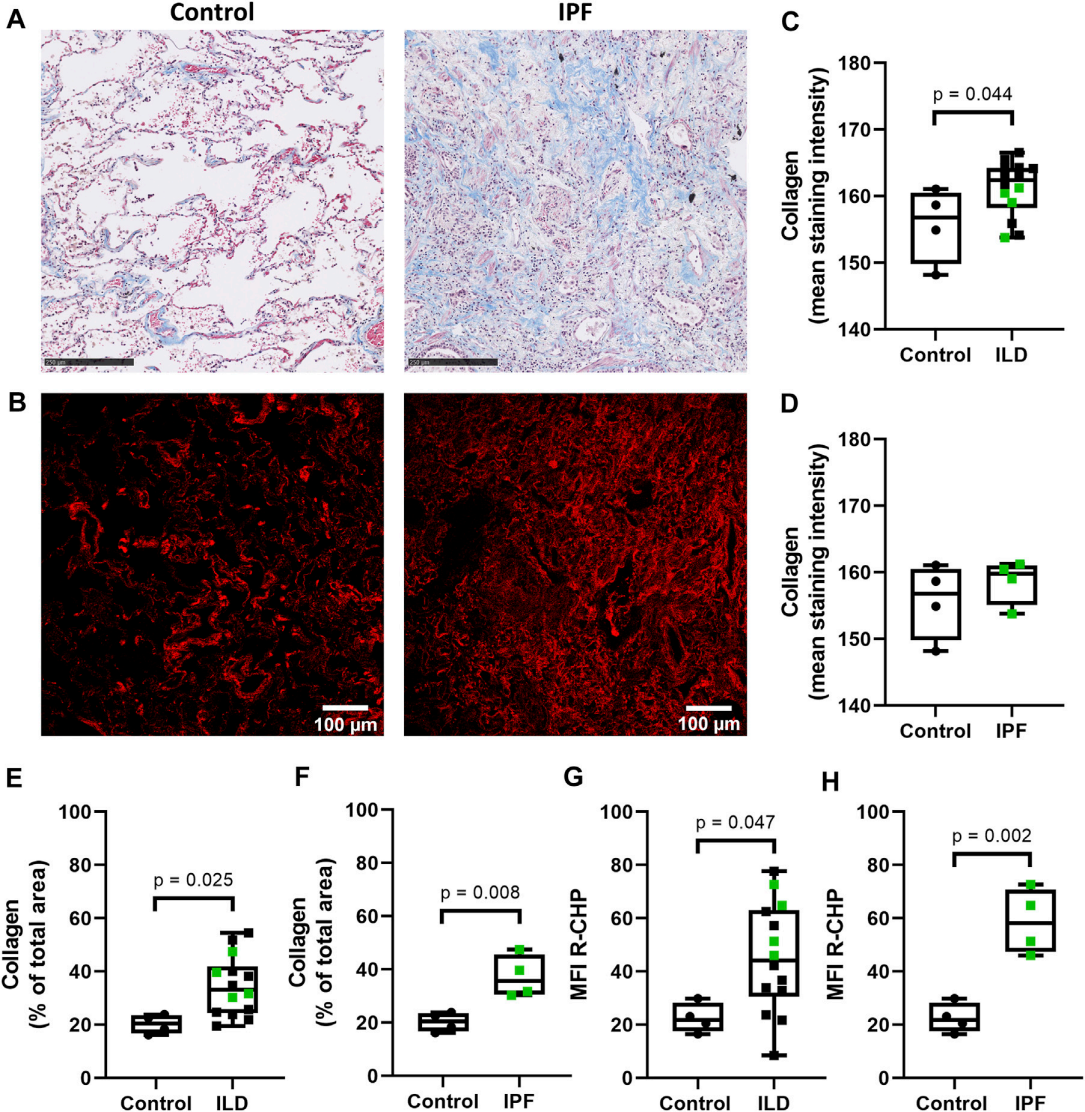
Higher levels of denatured collagen in lung tissue from patients with IPF. Sections of lung tissue were stained using Masson’s trichrome stain or Cy3-conjugated collagen hybridizing peptide (R-CHP). **(A)** Representative images of Masson’s trichrome stain in control lung tissue (left panel) and lung tissue of a patient with idiopathic pulmonary fibrosis (IPF, right panel), showing collagen fibers in blue and cytoplasm in red. Magnified part of the whole tissue section. Scale bar = 250 µm. **(B)** Representative fluorescence images of the R-CHP signal in control lung tissue (left panel) and lung tissue of a patient with IPF (right panel), from the same patients as in **(A)**. **(C-F)** Quantification of Masson’s trichrome staining, showing the mean staining intensity **(C,D)** and the relative amount of collagen in the whole imaging area **(E,F)**. Control lung tissue compared to ILD in general **(C,E)**, as well as specifically to IPF lung tissue [**(D,F)**, data from **(C,E)** partially repeated for visualization purposes]. **(G,H)** Quantification of the R-CHP signal, showing the average mean fluorescence intensity of at least three images per section. Control lung tissue compared to ILD in general **(G)**, as well as specifically to IPF lung tissue [**(H)**, data from **(G)** partially repeated for visualization purposes]. One experimental replicate per subject, sample size *n* = 4 (control) and *n* = 14 (ILD, including 4 lung tissues from patients with IPF). Data represented as min to max box-and-whiskers plots showing all points. Groups were compared using an unpaired *t*-test after assessing the normal distribution with use of a QQ plot and a Shapiro-Wilk normality test (Supplementary Figure 2A). *p* < 0.05 was considered significant.

To examine the levels of denatured collagen (i.e. collagen structural integrity) in lung tissue from control subjects and patients with ILD (including IPF), we stained lung tissue sections with the Cy3-conjugated collagen hybridizing peptide (R-CHP). As a reference, the total amount of collagen (both intact and denatured) was visualized with Masson’s trichrome stain (Figure 1A), indicating higher staining intensity and relative amount of collagen in the whole imaging area in ILDs compared to control tissues (Figures 1C,E). The staining intensity of total collagen was not different when comparing only the IPF lung tissues to the control lung tissue (Figures 1A,D), but the relative amount of collagen was higher in IPF tissue compared to control lung tissue (Figure 1F). The R-CHP staining visualized low levels of denatured collagen in control lung tissue (Figure 1B, left panel), but a wider spread and denser R-CHP signal was observed in lung tissue from patients with ILD and especially in IPF (Figure 1B, right panel). Quantification of the R-CHP signal indicated higher levels of denatured collagen in fibrotic lung tissue (Figure 1G) and especially in IPF lung tissue (Figure 1H), as compared to control lung tissue.

### Preservation of Idiopathic Pulmonary Fibrosis-Related Higher Levels of Denatured Collagen After Decellularization

**FIGURE 2:**
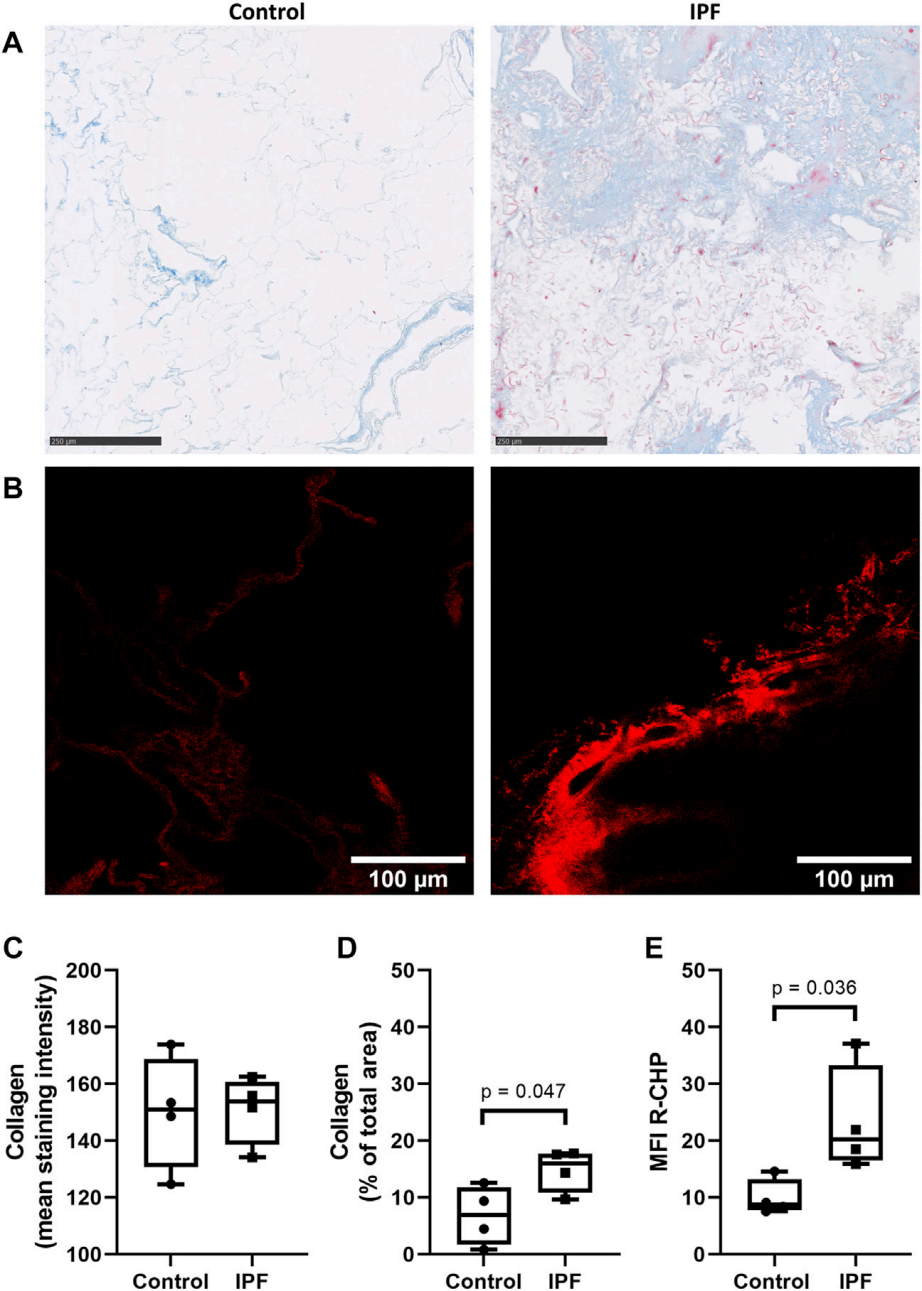
Preservation of IPF-related difference in levels of denatured collagen after decellularization of lung tissue. Decellularized lung tissue was stained using Masson’s trichrome staining or Cy3-conjugated collagen hybridizing peptide (R-CHP). **(A)** Representative images of Masson’s trichrome stain in control lung tissue (left panel) and lung tissue of a patient with idiopathic pulmonary fibrosis (IPF, right panel), showing collagen fibers in blue and cytoplasm in red. Magnified part of the whole decellularized tissue section. Scale bar = 250 µm. **(B)** Representative fluorescence images of R-CHP signal in decellularized lung tissue from control patients (left panel) and from patients with idiopathic pulmonary fibrosis (IPF, right panel). **(C,D)** Quantification of the Masson’s trichrome staining, showing the mean staining intensity **(C)** and the relative amount of collagen in the whole imaging area **(D)**. **(E)** Quantification of the R-CHP signal, showing the average mean fluorescence intensity of at least three images per decellularized lung tissue. One experimental replicate per subject, sample size *n* = 4 for both control and IPF decellularized lung matrices. Data represented as min to max box-and-whiskers plots showing all points. Groups were compared using an unpaired *t*-test after assessing the normal distribution with use of a QQ plot and a Shapiro-Wilk normality test (Supplementary Figure 2A). *p* < 0.05 was considered significant.

To verify whether the difference in collagen denaturation was preserved after decellularization of lung tissue, decellularized control and fibrotic lung tissue were also stained using the same collagen hybridizing peptide and Masson’s trichrome stain. Decellularized lung tissue from patients with IPF had a higher amount of collagen, measured as percentage of the imaged area (Figure 2D), but did not show higher staining intensity of collagen when compared to control lung tissue (Figures 2A,C). However, more R-CHP signal was observed in decellularized lung tissue from patients with IPF (Figure 2B, right panel) than in control lung tissue (Figure 2B, left panel) and quantification confirmed higher levels of denatured collagen in IPF decellularized lung tissue compared to control (Figure 2E). The fibrotic decellularized lung tissue contained approximately twice as much signal as the control decellularized lung tissue, reflecting the pattern observed in the native lung tissue. This indicates that the collagen denaturation status is retained in decellularized lung tissue, confirming its suitability for studying cell-(IPF) matrix interactions.

### Lung Fibroblasts Deposit More Extracellular Matrix When Seeded in Idiopathic Pulmonary Fibrosis-Derived Lung Matrices

**FIGURE 3:**
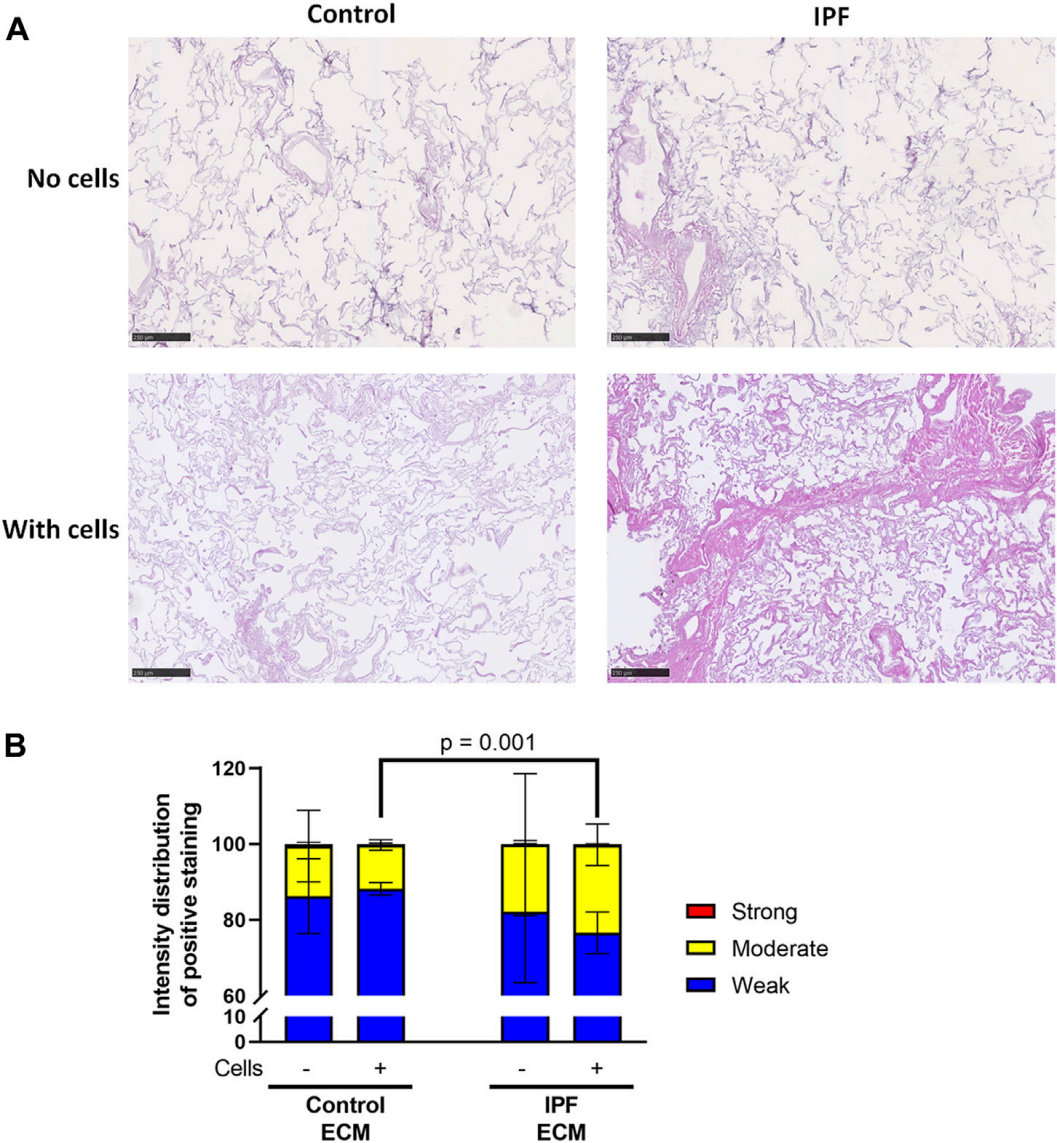
More ECM deposition in IPF matrices upon recellularization compared to control matrices. Primary lung fibroblasts were seeded into decellularized matrices and cultured for 7 days. Sections of recellularized lung tissue were stained with haematoxylin (blue) and eosin (pink). **(A)** Representative images of the stained recellularized matrices, showing a magnified part of the image of the total tissue section. Scale bar = 250 μm. **(B)** Quantification of the intensity distribution of the staining. Groups were compared using a two-way ANOVA followed by the original FDR method of Benjamin and Hochberg post hoc test. *p* < 0.05 was considered significant. All matrices are derived from the same control and IPF donor, primary lung fibroblasts were obtained from three different control donors.

Primary lung fibroblasts were seeded into the decellularized matrices and cultured for 7 days to investigate the effects of the altered matrix in IPF on cellular behavior. Total deposition of ECM by the lung fibroblasts was visualized by H&E staining of the recellularized tissue (Figure 3A). Before recellularization, unseeded matrices showed variable, but similar tissue staining, indicating the heterogeneity of IPF (Supplementary Figure 3A). After recellularization, more tissue staining and denser tissue areas, reflecting the characteristics of the fibrotic foci in IPF, were observed in the IPF matrix compared to the control matrix (Figures 3A, 5B). As the IPF matrix with cells showed both dense and more open tissue areas, illustrating the heterogeneous nature of the tissue response in IPF, the intensity distribution of the staining was analyzed. The outcome of this analysis provides an indication of the relative amount of dense tissue in a section: areas with higher staining intensity often consist of denser tissue. After recellularization, IPF matrices had proportionally more moderate intensity staining and less weak intensity staining than control matrices (Figure 3B), indicating that more dense tissue areas are present in recellularized IPF matrices than in control matrices. Comparable results were observed when extending the culture time up to 14 days (data not shown). Hence, lung fibroblasts remodel more ECM with more dense areas when seeded in IPF-derived lung matrices compared to control matrices.

### Lung Fibroblasts Equalize the Levels of Denatured Collagen in Control and Idiopathic Pulmonary Fibrosis Lung Matrices

**FIGURE 4:**
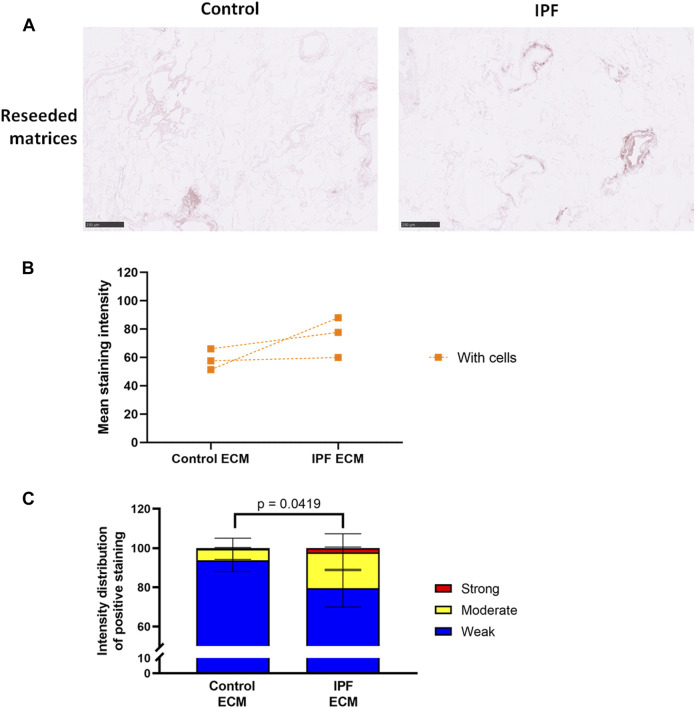
Equal levels of denatured collagen in recellularized control and IPF matrices. Primary lung fibroblasts were seeded into decellularized matrices and cultured for 7 days. Sections of recellularized lung tissue were stained using collagen hybridizing peptide (B-CHP, brown) and an eosin counterstain (pink). **(A)** Representative images of the B-CHP stained recellularized matrices, showing a magnified part of the image of the total tissue section. Scale bar = 250 µm. **(B)** Quantification of the B-CHP staining, showing the mean staining intensity. Groups were compared using an unpaired *t* test after assessing the normal distribution with use of a QQ plot and a Shapiro-Wilk normality test (Supplementary Figure 2A). No significant differences were observed (*p* > 0.05). **(C)** Quantification of the intensity distribution of the B-CHP staining. Groups were compared using a two-way ANOVA followed by the original FDR method of Benjamini and Hochberg post hoc test. *p* > 0.05 was considered significant. All matrices are derived from the same control and IPF donor, primary lung fibroblasts were obtained from three different control donors (*n* = 3, dots connected per *n*).

To assess if more ECM deposition and dense tissue areas in IPF matrices corresponded with an alteration in collagen denaturation status, the levels of denatured collagen after recellularization were visualized using a biotin-conjugated collagen hybridizing peptide (B-CHP) (Figure 4A; Supplementary Figure 3B). Interestingly, the differences in the level of denatured collagen seen between control and IPF matrices (Figure 2) were no longer evident after reseeding (Figure 4B). Still, more moderate and less weak CHP staining was detected in the recellularized IPF matrices compared to the recellularized control matrices (Figure 4C). This observation suggests that fibroblasts were able to remodel the degree of denatured collagen in both control and IPF matrices, but were less effective in doing so in the IPF matrices.

### Periostin and Fibronectin Do Not Contribute to the Higher Levels of Extracellular Matrix Deposition

As no differences were observed in the levels of denatured collagen between control and IPF matrices after recellularization, we investigated whether the ECM proteins fibronectin or periostin were contributing to the higher levels of ECM in IPF matrices. However, periostin and fibronectin levels did not change in the recellularized IPF matrices when compared to recellularized control matrices (Supplementary Figures 4, 5). Thus, changes in the levels of periostin or fibronectin did not play a key role in regulating the altered matrix deposition in the IPF matrices observed in this study.

### Adipose Tissue-Derived Stromal Cell Conditioned Medium Attenuates Extracellular Matrix Deposition by Lung Fibroblasts in Lung Matrices

**FIGURE 5:**
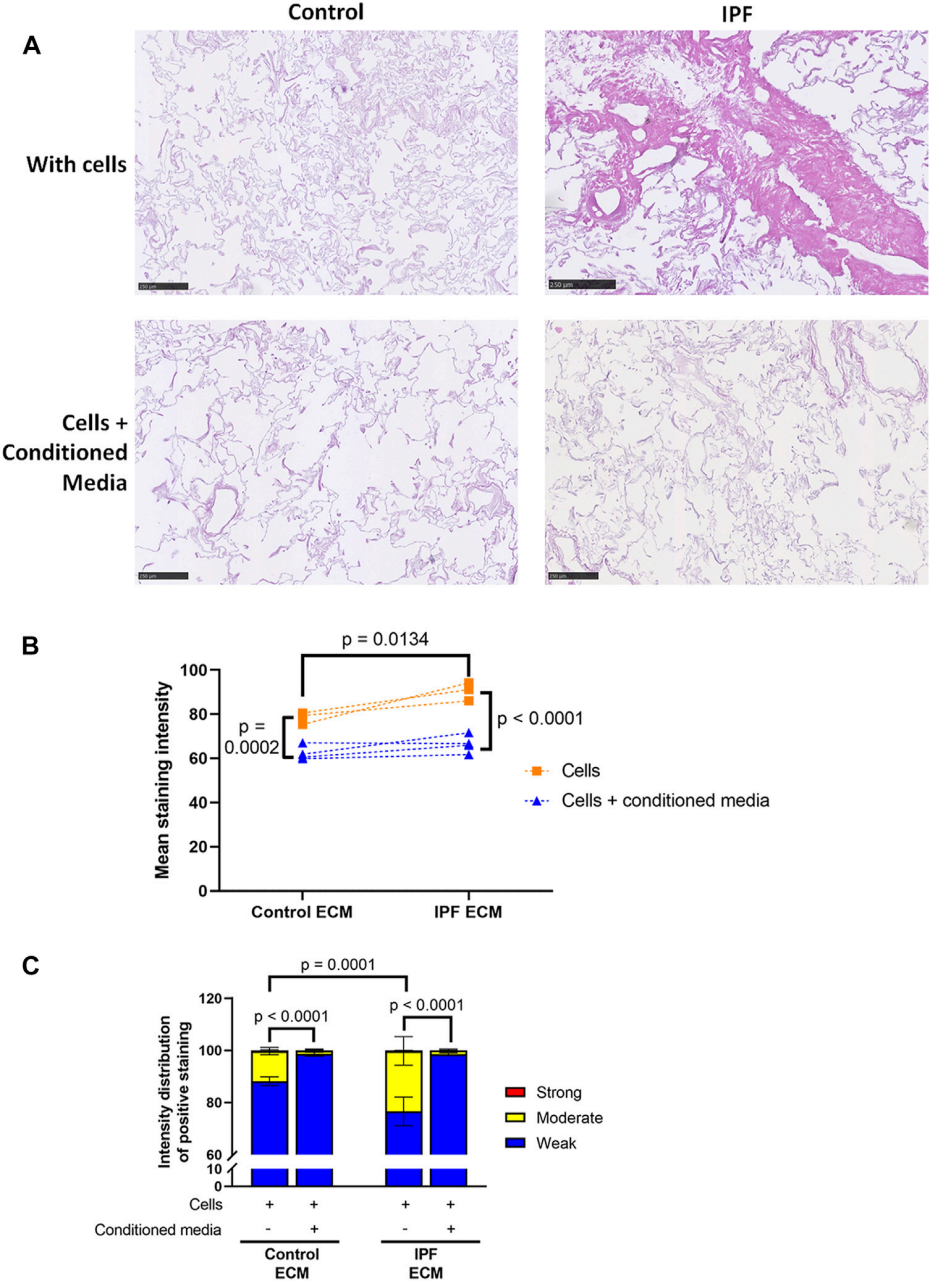
Less ECM deposition in recellularized control and IPF matrices upon ASC-CM treatment. Primary lung fibroblasts were seeded into decellularized matrices and cultured in the absence or presence of ASC-CM for 7 days. Sections of recellularized lung tissue were stained with haematoxylin (blue) and eosin (pink). **(A)** Representative images of the stained recellularized matrices, showing a magnified part of the whole tissue section. Scale bar = 250 μm. **(B)** Quantification of the H&E staining, showing the mean staining intensity. **(C)** Quantification of the intensity distribution of the staining. Groups were compared using a two-way ANOVA followed by the original FDR method of Benjamini and Hochberg as a post-hoc test. *p* < 0.05 was considered significant. All matrices are derived from the same control and IPF donor, primary lung fibroblasts were obtained from three different control donors (*n* = 3, dots connected per *n*).

To study the potential antifibrotic effect of ASC-CM in this human *in vitro* model of IPF, decellularized matrices were reseeded with primary human lung fibroblasts and incubated with or without ASC-CM for 7 days. The addition of ASC-CM clearly resulted in less total ECM in both control and IPF matrices, as visualized by an H&E staining (Figure 5A). Quantification indeed revealed a drop in mean staining intensity (*p* = 0.0002, Figure 5B), as well as changes in the staining intensity distribution between recellularized matrices in the presence and absence of ASC-CM (Figure 5C). ASC-CM treatment resulted in a shift from moderate intensity staining to weak intensity staining in both IPF and control matrices when compared to untreated reseeded matrices (Figure 5C). The higher levels of weak staining indicate that there is less dense tissue in the ASC-CM treated matrices compared to both IPF and control matrices without ASC-CM. Also, the significant differences in total tissue and relative amount of dense tissue areas between control and IPF recellularized matrices (shown in Figure 3B) were abolished by ASC-CM treatment. Thus, even though ECM deposition and tissue density were higher in recellularized IPF matrices compared to control matrices, the addition of ASC-CM neutralized the profibrotic signals provided to the fibroblasts by the ECM, thereby resulting in less ECM remodeling and lower tissue density to similar levels in control and IPF matrices.

### Adipose Tissue-Derived Stromal Cell Conditioned Medium Does Not Influence the Levels of Denatured Collagen in Recellularized Lung Matrices

**FIGURE 6:**
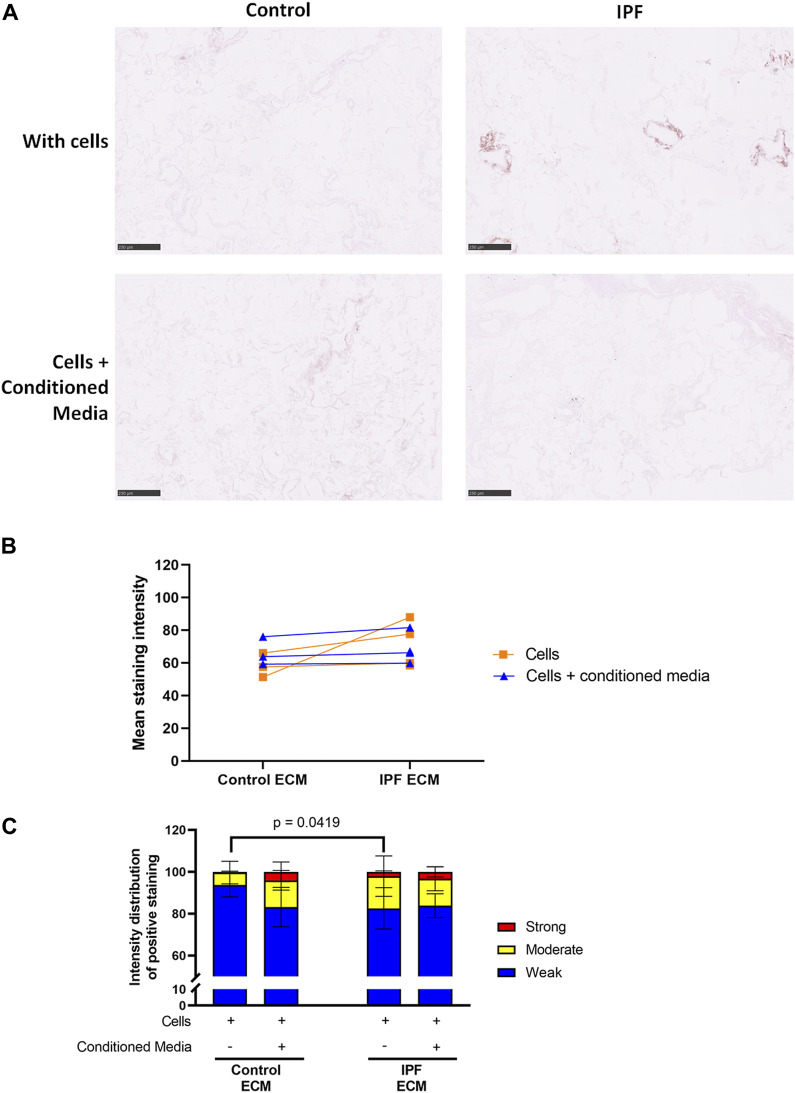
Treatment with ASC-CM does not alter the levels of denatured collagen. Primary lung fibroblasts were seeded into decellularized matrices and cultured in the absence or presence of ASC-CM for 7 days. Sections of recellularized lung tissue were stained using collagen hybridizing peptide (B-CHP, brown) and an eosin counterstain (pink). Data of matrices with cells are repeated from Figure 4 to allow for a clear comparison. **(A)** Representative images of the stained recellularized matrices showing a magnified part of the image of the total tissue section. Scale bar = 250 μm. **(B)** Quantification of the B-CHP staining, showing the mean staining intensity. **(C)** Quantification of the intensity distribution of the B-CHP staining. Groups were compared using a two-way ANOVA followed by the original FDR method of Benjamini and Hochberg as a post-hoc test. *p* < 0.05 was considered significant. All matrices are derived from the same control and IPF donor, primary lung fibroblasts were obtained from three different control donors (*n* = 3, dots connected per *n*).

The levels of denatured collagen after ASC-CM treatment were assessed with a B-CHP staining. The representative images, mean staining intensity and the distribution of staining intensity did not show significant alterations in levels of denatured collagen upon ASC-CM treatment in comparison to recellularized matrices in the absence of ASC-CM (Figure 6). This indicates that addition of ASC-CM does not interfere with the collagen remodeling activity of primary lung fibroblasts. Similarly, the levels of periostin and fibronectin were also not altered by ASC-CM treatment (Supplementary Figures 4, 5).

## Discussion

In this study, we found that ASC-CM can inhibit IPF lung matrix-driven ECM deposition by primary human lung fibroblasts. While higher levels of denatured collagen were observed in both native and decellularized lung tissue from patients with IPF than in control lung tissue, this characteristic was independent of the factors driving the enhanced matrix deposition by lung fibroblasts in IPF matrices. Reseeding with human lung fibroblasts reversed the collagen denaturation status of the matrices, bringing the denatured collagen in both control and IPF matrices to comparable levels. We investigated the potential regulatory role of fibronectin and periostin within the matrices but found that these levels were not altered, making these proteins unlikely candidates as the factors responsible for the modulation of the fibroblast responses.

There is a lot of interest in the potential of stem cells as a therapeutic approach for lung regeneration in IPF. To date, the impact of these cells (or their bioactive secreted factors) on the interactions of resident cells with the fibrotic microenvironment, and more importantly vice versa, had not been elucidated yet. In light of this, we investigated whether ASC-CM could interfere with fibroblast-(IPF) matrix interactions and subsequent ECM deposition and remodeling. Although ASC-CM did not modulate levels of denatured collagen, fibronectin or periostin, it did result in less ECM deposition in both control and IPF-derived decellularized matrices, thus demonstrating an antifibrotic effect of the ASC-CM. The lack of influence on the denatured collagen may reflect the fact that cells establish an equilibrium achieving a certain degree of spatial arrangement within the collagen structural organization and that further collagen remodeling is not required. Further investigation, beyond the scope of this study, is required to confirm this hypothesis. The abrogation of tissue remodeling induced by the ASC-CM is in accordance with animal models of IPF, in which ASC-CM administration also attenuated lung fibrosis (Rathinasabapathy et al., 2016; Felix et al., 2020). Of note, it is of interest that ASC-CM treatment not only contributed to less total ECM deposition in IPF matrices, but also to a similar level in control matrices, again suggesting that there is an equilibrium state for the collagen fiber organization that the cells seek to achieve. These data illustrate that ASC-CM generally inhibits ECM deposition by fibroblasts, independent of the fibrotic status of the ECM. Although this *in vitro* study indicates that conditioned media from stem cells could have a therapeutic effect in IPF, extensive (clinical) validation and safety studies are needed before considering clinical application.

As this study aimed at investigating the effects of the fibrotic ECM and ASC-CM on fibroblast behavior, the standard cell culture media were adapted. Regular FBS in culture medium can activate fibroblasts and was therefore omitted, as it could have masked any effects from the ECM or the ASC-CM that we sought to investigate. Therefore, fibroblasts were cultured in the matrices in cell culture medium with 1% BSA instead of FBS. However, H&E staining of the matrices indicated that no fibroblasts were left in our matrices after 7 days incubation. A limitation of our study could be that this 1% BSA-containing medium was not an optimal culture condition for the fibroblasts. However, our results illustrate that the fibroblasts were able to remodel their microenvironment during the period they were present in the lung matrices.

The secretome of ASCs consists of a multitude of factors, which are involved in different processes such as cell proliferation and angiogenesis (Salgado et al., 2010; Kapur and Katz, 2013). This is the main advantage of ASC-CM: instead of administering a single factor that could potentially inhibit fibrosis, multiple factors are administered that can have synergistic effects. We have shown previously that our ASC-CM inhibits TGF-β1-induced differentiation of human dermal fibroblasts to myofibroblasts (Spiekman et al., 2014). In the current study, IPF ECM induced a profibrotic phenotype in lung fibroblasts, as measured by higher levels of ECM deposition. ASC-CM treatment inhibited this profibrotic phenotype, allowing less ECM deposition in its presence. Interestingly, in recent studies, ASCs in culture were stimulated with either pro-inflammatory factors or a specific microRNA (miR-146a) resulting in a CM that was more anti-inflammatory, pro-angiogenic or aided in homing of immune cells to tumors (Domenis et al., 2018; Li et al., 2019; Waters et al., 2019). To further enhance the antifibrotic properties of ASC-CM, addition of specific factors to ASC cultures to precondition these cells could be an option.

To investigate the effect of ECM on the remodeling behavior of fibroblasts, we visualized collagen denaturation in IPF and control matrices. The presence of higher levels of structurally disrupted collagen in lung tissue from patients with IPF indicates that even though there is excessive accumulation of collagen, collagen degradation is still taking place at the molecular level. This is, to our knowledge, the first study to examine the degree of collagen denaturation in human lung tissues. Interestingly, the comparable levels of denatured collagen in IPF and control matrices after cell seeding indicate that fibroblasts were able to repair or remove excess denatured collagen found in IPF lung matrices. The cells sensed their microenvironment and remodeled this as such to a defined level that was equivalent across the two different starting matrices. In contrast to our observations regarding cellular remodeling of denatured collagen, cellular responses to decellularized scaffolds differed between fibrotic and nonfibrotic matrices. We found that fibrotic matrix stimulated fibroblasts to produce and deposit more ECM, confirming the feedback loop in fibrotic matrices that has been observed previously (Booth et al., 2012; Parker et al., 2014).

Decellularized (lung) matrices are gaining attention in tissue engineering as *in vitro* models to study cellular behavior (Gilpin and Wagner, 2018; Taylor et al., 2018). However, it is important to keep in mind that decellularization protocols can affect the mechanical and morphological properties of the ECM (Petersen et al., 2012; Hwang et al., 2017b). The collagen hybridizing peptide used in this study has previously been applied to show that decellularizing agents can induce collagen denaturation (Hwang et al., 2017b). In this study, we could not investigate the effect of our decellularization protocol on the absolute levels of denatured collagen, as the native and decellularized lung tissues were not derived from the same patient and processed differently. Nonetheless, we did observe that the relative difference in levels of denatured collagen between lung tissue from patients with IPF and controls was comparable in native and decellularized lung tissue. The preservation of this IPF-related difference in levels of denatured collagen emphasizes that decellularized lung tissue is suitable for *in vitro* models studying cellular responses to this denatured collagen*.*

A major asset of our study is the use of human materials. Although bleomycin-induced pulmonary fibrosis is currently the most accepted model to investigate IPF, the different etiology, disease development, and especially the spontaneous fibrosis resolution after a period of time, are quite different from the human condition (Tashiro et al., 2017). Hence, by using human materials, we represent human disease specifically. However, this approach also generates several challenges. IPF is associated with a very heterogeneous pattern, with both control and fibrotic areas being recognized within lung tissue of the same patient (Travis et al., 2002; DePianto et al., 2015). This likely contributes to the significant variation in levels of denatured collagen observed between IPF samples. Interpatient variability in disease severity could also contribute to this variation (Raghu et al., 2011). In liver fibrosis, collagen hybridizing peptide staining has recently been suggested to have value in quantitatively assessing disease severity and predicting the need for liver transplantation (Jaramillo et al., 2020).

In conclusion, this study has shown that levels of denatured collagen are higher in lung tissue from patients with IPF than from control patients and that this difference is preserved in decellularized lung tissue. After recellularization, IPF ECM drives fibroblasts toward profibrotic behavior, which can be counteracted by administration of ASC-CM. These findings further confirm in a human *in vitro* model that the use of ASC-CM may be a promising approach to stop fibrotic ECM from directing profibrotic behavior in fibroblasts in IPF.

## Data Availability

The raw data supporting the conclusion of this article will be made available by the authors upon reasonable request, without undue reservation.
