# Supplementary material for: Adipose Stromal Cell-Secretome Counteracts Profibrotic Signals From IPF Lung Matrices
Source: Front Pharmacol. 2021 Jul 28;12:669037. doi: 10.3389/fphar.2021.669037 (PMC8355988; doi:10.3389/fphar.2021.669037)
Supplement: Supplementary file 1 [file DataSheet1.docx]

**Supplementary Information**

**Supplementary Materials and Methods**

*Immunohistochemistry*

Antigen retrieval was performed by microwaving the slides for 15 minutes in stain-specific buffer. Tris/ethylene-diamine-tetraacetic acid (Tris/EDTA) buffer and EDTA buffer were used for the fibronectin and periostin staining, respectively. Endogenous peroxidases were blocked by incubating the slides for 30 minutes in PBS with 0.3% H_2_O_2_. Slides were washed in PBS and incubated with anti-periostin antibody (Abcam, Cambridge, UK) in a 1:100 dilution in 1% BSA/PBS or anti-fibronectin-1 antibody (Abcam, Cambridge, UK) in a 1:200 dilution in 1% BSA/PBS for 1 hour. The secondary antibody was incubated for 30 minutes in a 1:100 dilution in 1% BSA/PBS (Goat-anti-rabbit peroxidase (GAR^PO^), p0448, Agilent technologies (Dako), Santa Clara, USA). Slides were washed in PBS and the third antibody was added. This third antibody (Rabbit-anti-goat peroxidase (RAG^PO^), p0449, Agilent technologies (Dako), Santa Clara, USA) was incubated for 30 minutes in a 1:100 dilution in 1% BSA/PBS. Diaminobenzidine (DAB) substrate (Sigma, St Louis, USA) was mixed with PBS containing 0.03% H_2_O_2_. The slides were incubated for 10 minutes in this DAB-H_2_O_2_ mix. A counterstain with eosin (Chroma, Bellows Falls, USA) was performed and slides were dehydrated and mounted in Tissue-Tek Coverslipping Film with the Sakura Tissue-Tek Film Coverslipper.

**Supplementary Figures**

**
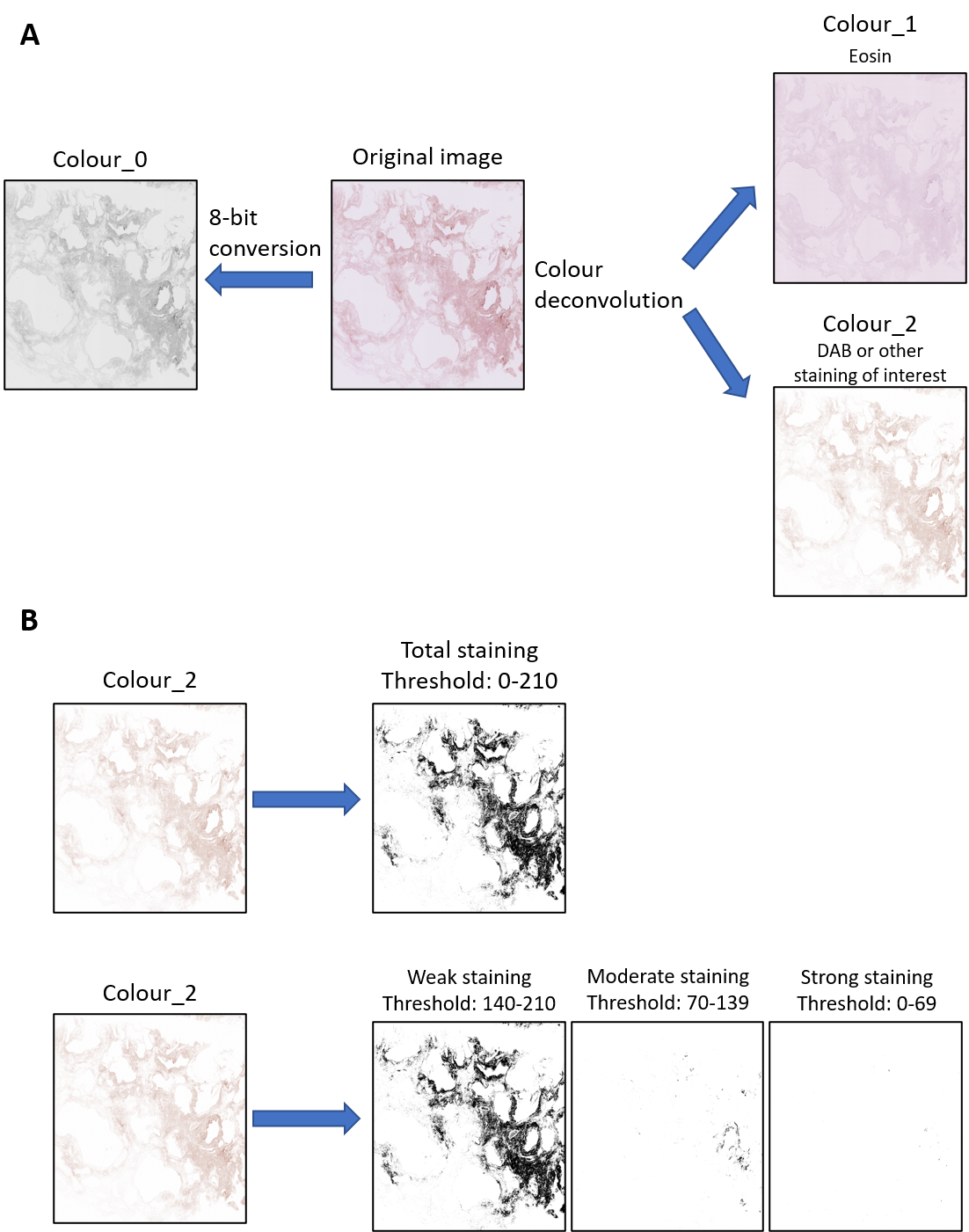
**

**Supplementary figure 1. Workflow for analysis of staining intensity with weak/moderate/strong distribution.** Original images were obtained by scanning the microscope slide with a slide scanner. (**A**) Images were converted to 8-bit for total tissue area calculation (colour_0). Simultaneously, the original image was deconvoluted to separate the colors (colour_1 (eosin) and colour_2 (stain of interest (DAB))). (**B**) The staining of interest (colour_2) was analyzed for total area stained and intensity of stain of positive pixels.  The intensity of positive pixels was separated into relative amounts of weak, moderate and strong staining, calculated using three tertiles within the total threshold boundaries.


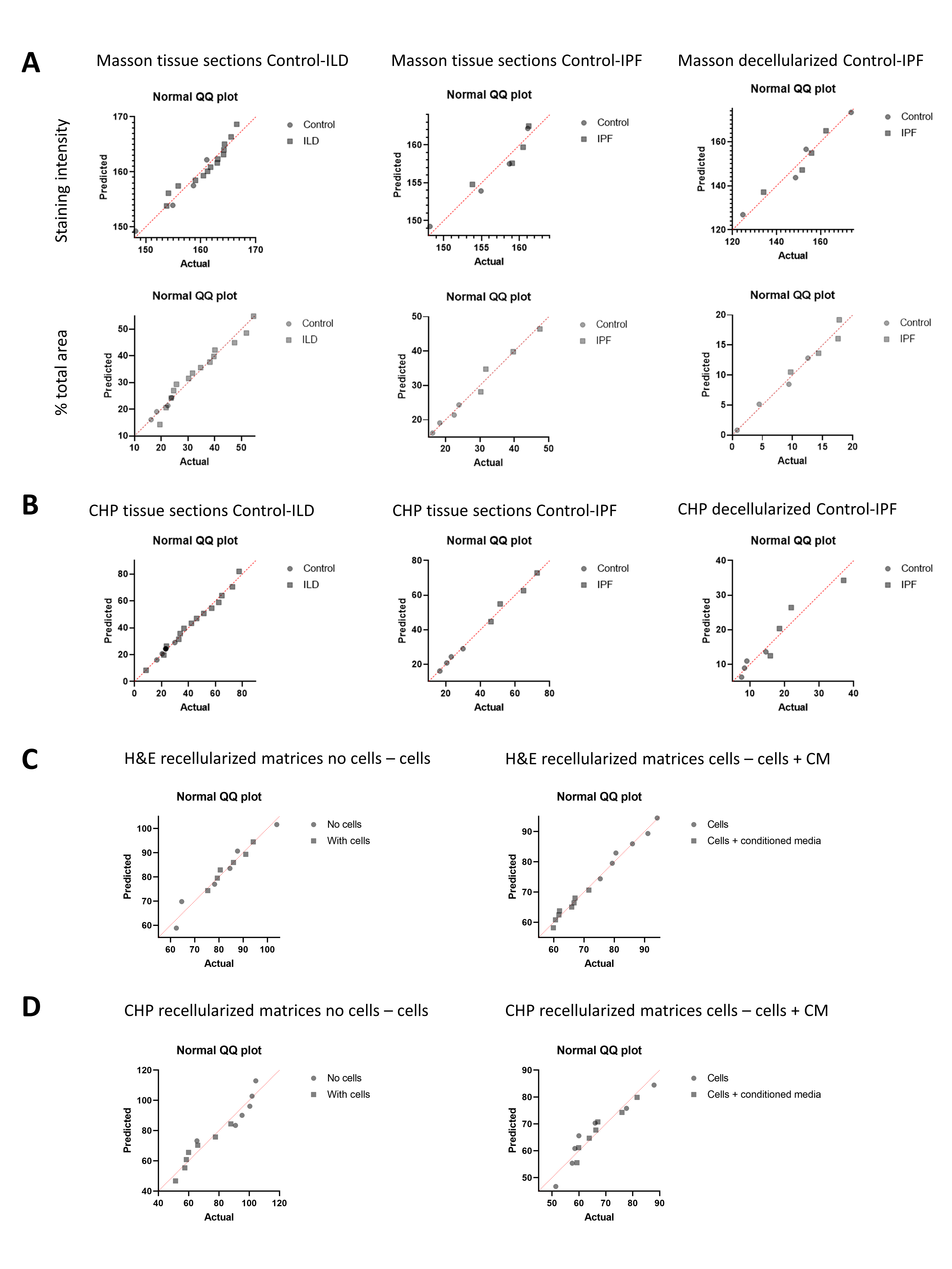


**Supplementary figure 2. QQ plots show normal distribution of data.** QQ plots were generated for all datasets to test normality. (**A)** QQ plots for Masson’s trichrome stain on whole lung tissues and decellularized lung tissues. Mean staining intensity and % collagen of total area. (**B)** QQ plots for CHP staining on whole lung tissues and decellularized lung tissues. (**C)** QQ plots for H&E staining of recellularized matrices. (**D)** QQ plots for CHP staining of recellularized matrices.

**
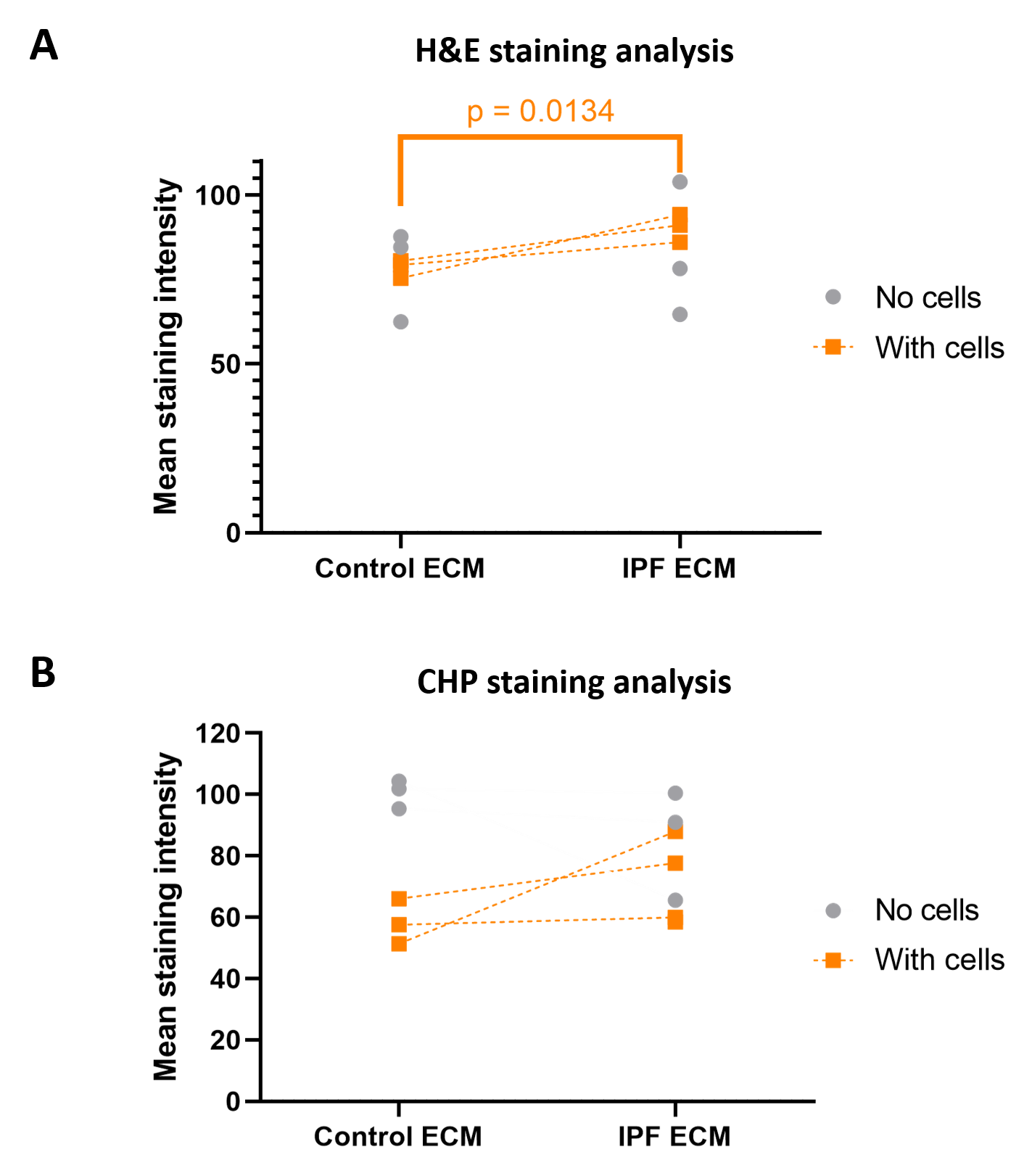
**

**Supplementary figure 3. Comparison of mean staining intensity of decellularized matrices without cells and after recellularization shows no significant differences.** Primary lung fibroblasts were seeded onto decellularized matrices and cultured for 7 days. Simultaneously, decellularized matrices without cells were cultured. Sections of recellularized lung tissue were stained with H&E or for CHP and counterstained with eosin (pink). Mean staining intensity of H&E and CHP staining was measured. Data with no cells were inconclusive. (**A)** Mean staining intensity of H&E staining. (**B)** Mean staining intensity of CHP staining. Groups were compared using a two-way ANOVA followed by the original FDR method of Benjamini and Hochberg post hoc test. P<0.05 was considered significant. All matrices are derived from the same control and IPF donor, primary lung fibroblasts were obtained from 3 different control donors (n=3).


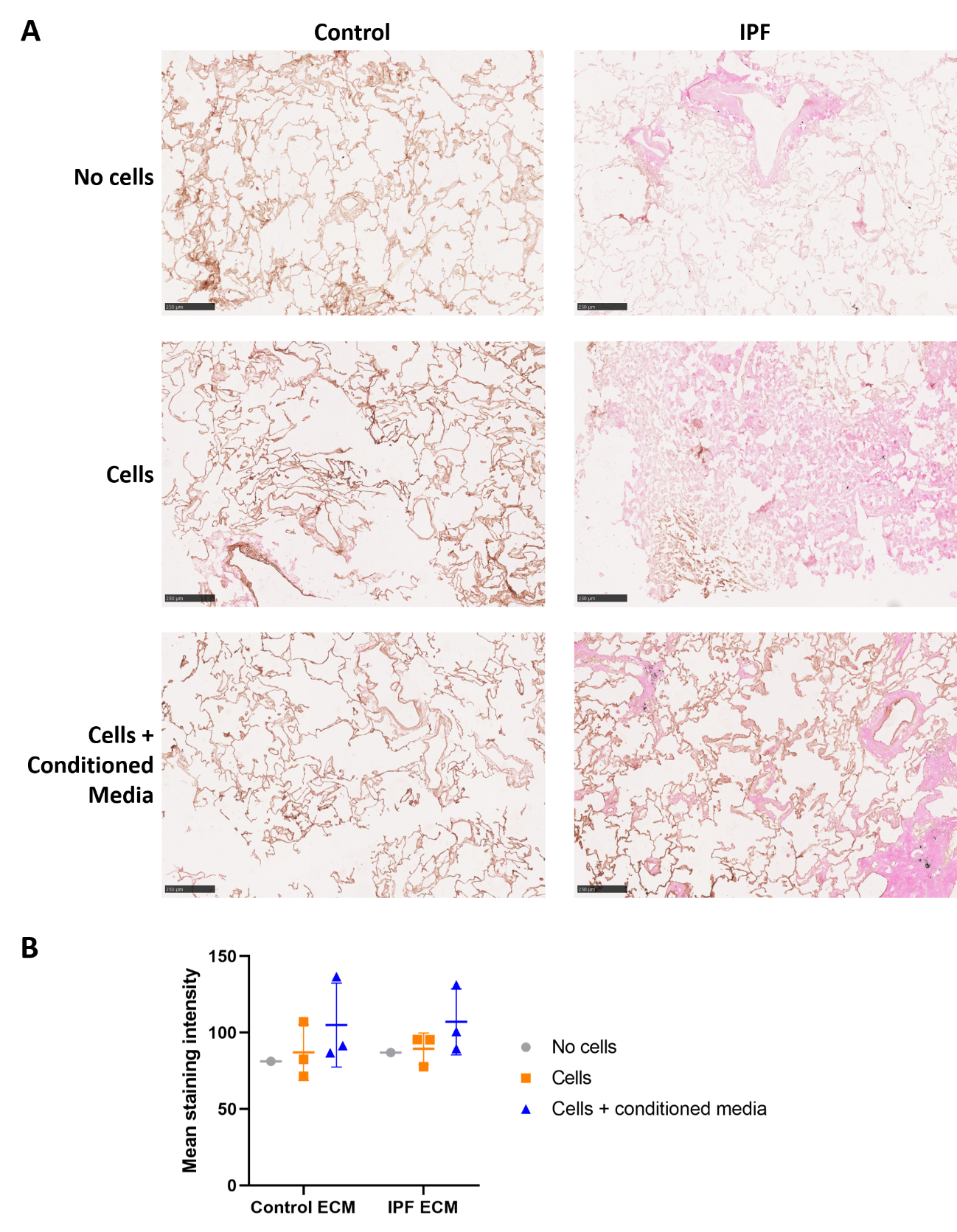


**Supplementary figure 4. Fibronectin expression does not change upon recellularization or ASC-CM treatment.** Primary lung fibroblasts were seeded into the decellularized matrices and cultured in the absence or presence of ASC-CM for 7 days. Sections of recellularized lung tissue were stained for fibronectin (brown) and counterstained with eosin (pink). (**A)** Representative images of the stained recellularized matrices. Scale bar = 250 μm. (**B)** Quantification of the fibronectin staining, showing the mean staining intensity. Groups were compared using a two-way ANOVA followed by a Tukey post-hoc test. No significant differences were observed (p>0.05). All matrices are derived from the same control and IPF donor, primary lung fibroblasts were obtained from 3 different control donors (n=3).


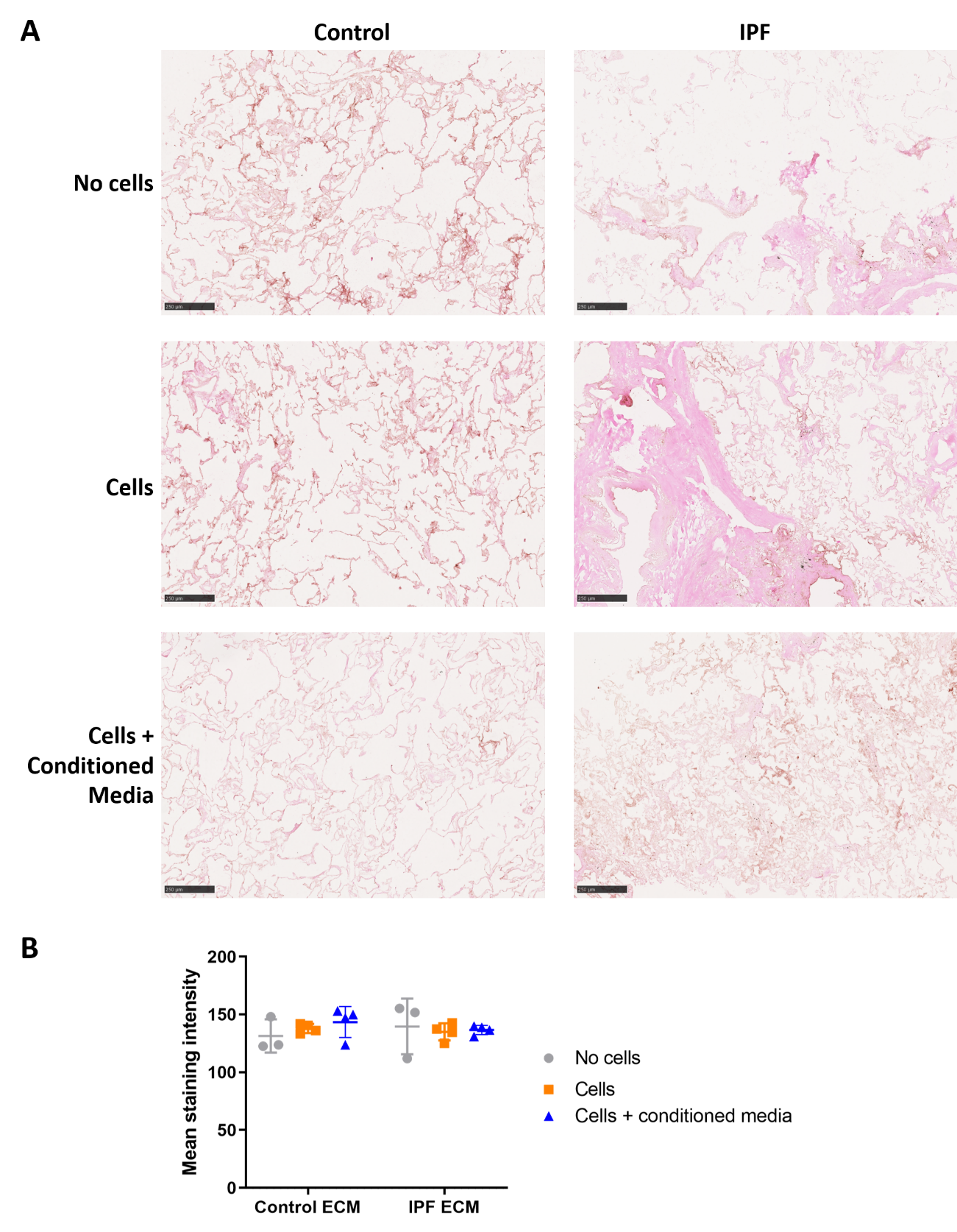


**Supplementary figure 5. Periostin expression does not change upon recellularization or ASC-CM treatment.** Primary lung fibroblasts were seeded into the decellularized matrices and cultured in the absence or presence of ASC-CM for 7 days. Sections of recellularized lung tissue were stained for periostin (brown) and counterstained with eosin (pink). (**A)** Representative images of the stained recellularized matrices. Scale bar = 250 μm. (**B)** Quantification of the Fibronectin staining, showing the mean staining intensity. Groups were compared using a two-way ANOVA followed by a Tukey post-hoc test. No significant differences were observed (p>0.05). All matrices are derived from the same control and IPF donor, primary lung fibroblasts were obtained from 3 different control donors (n=3).
